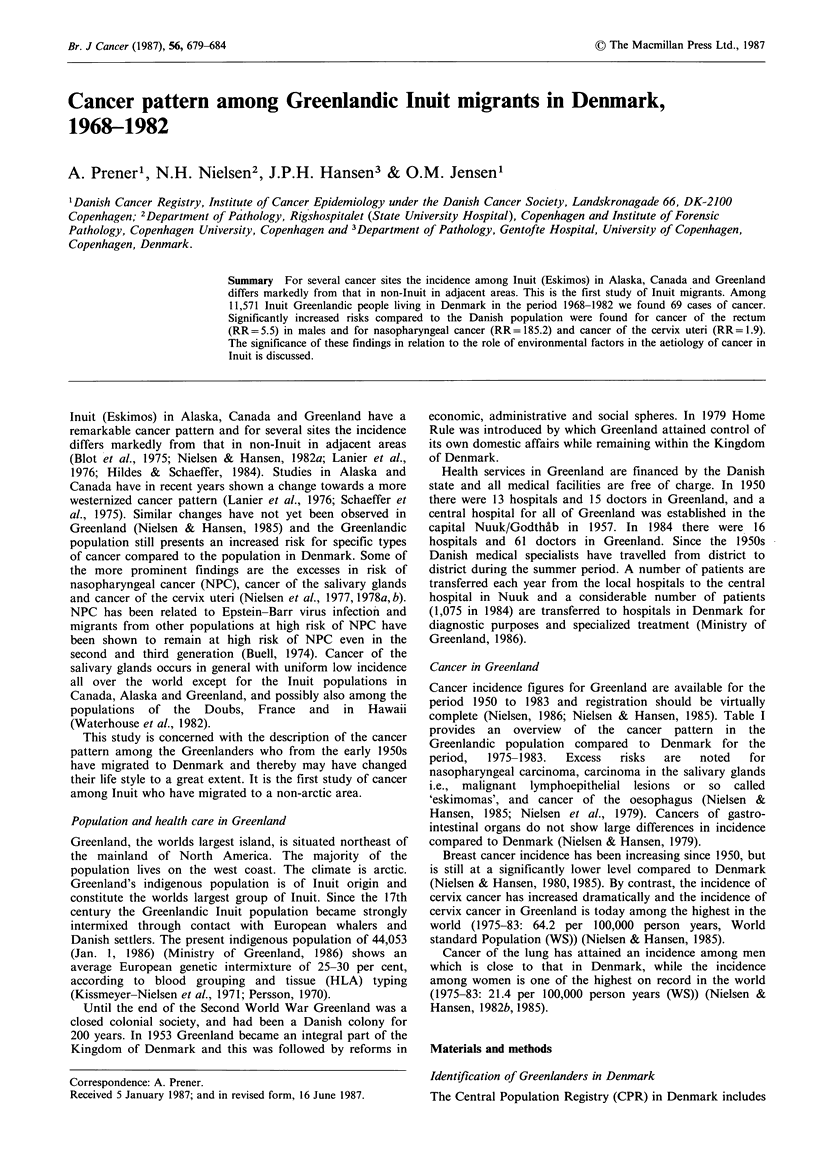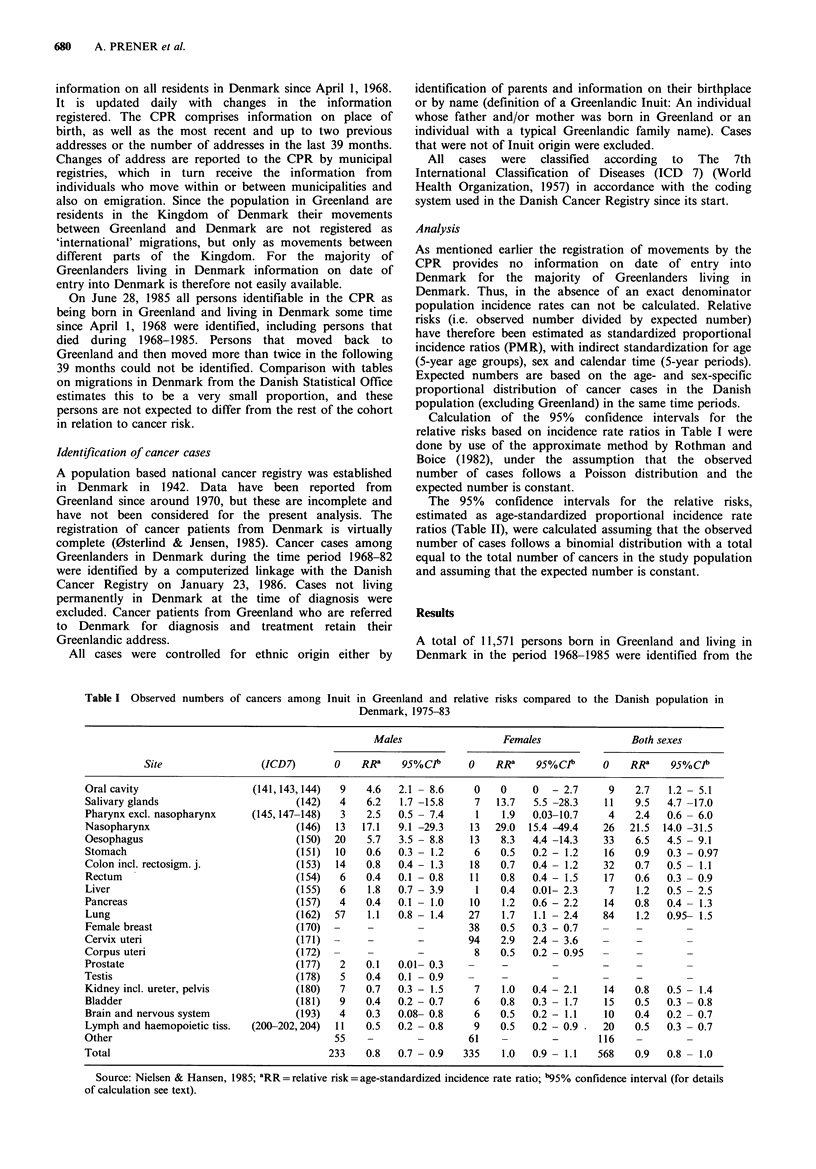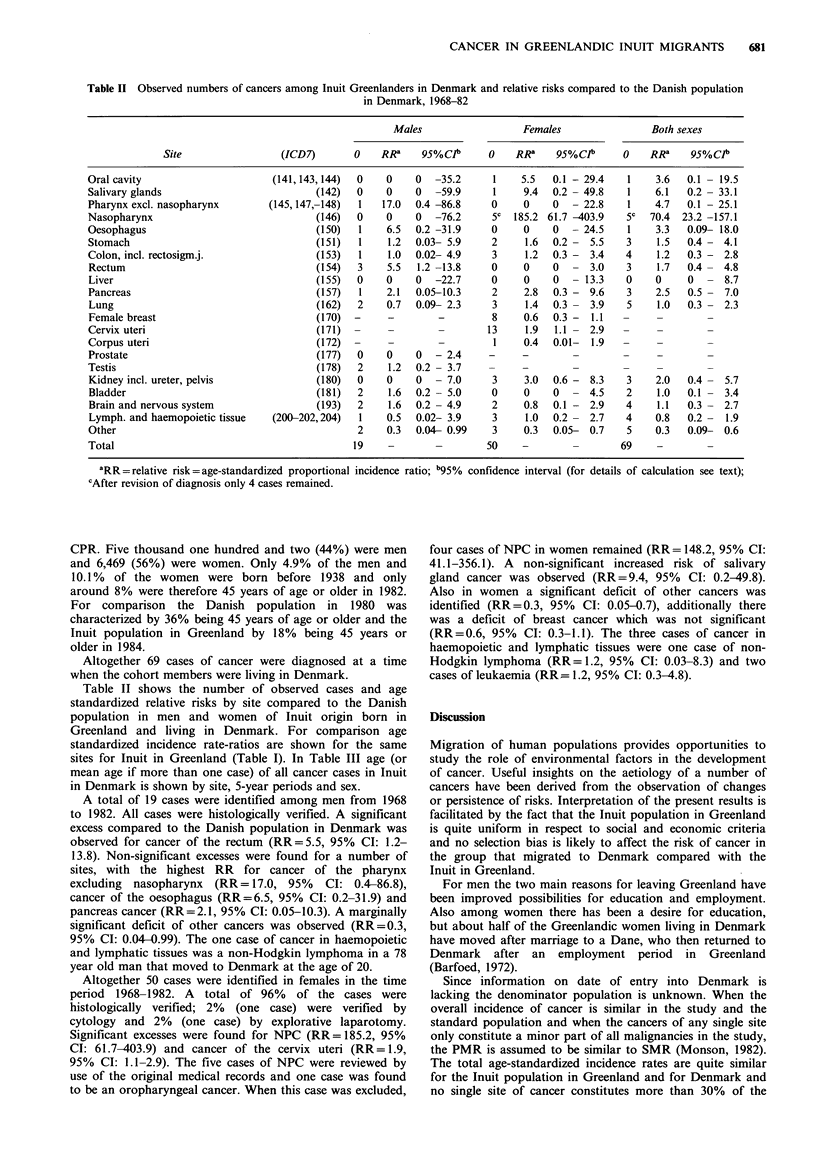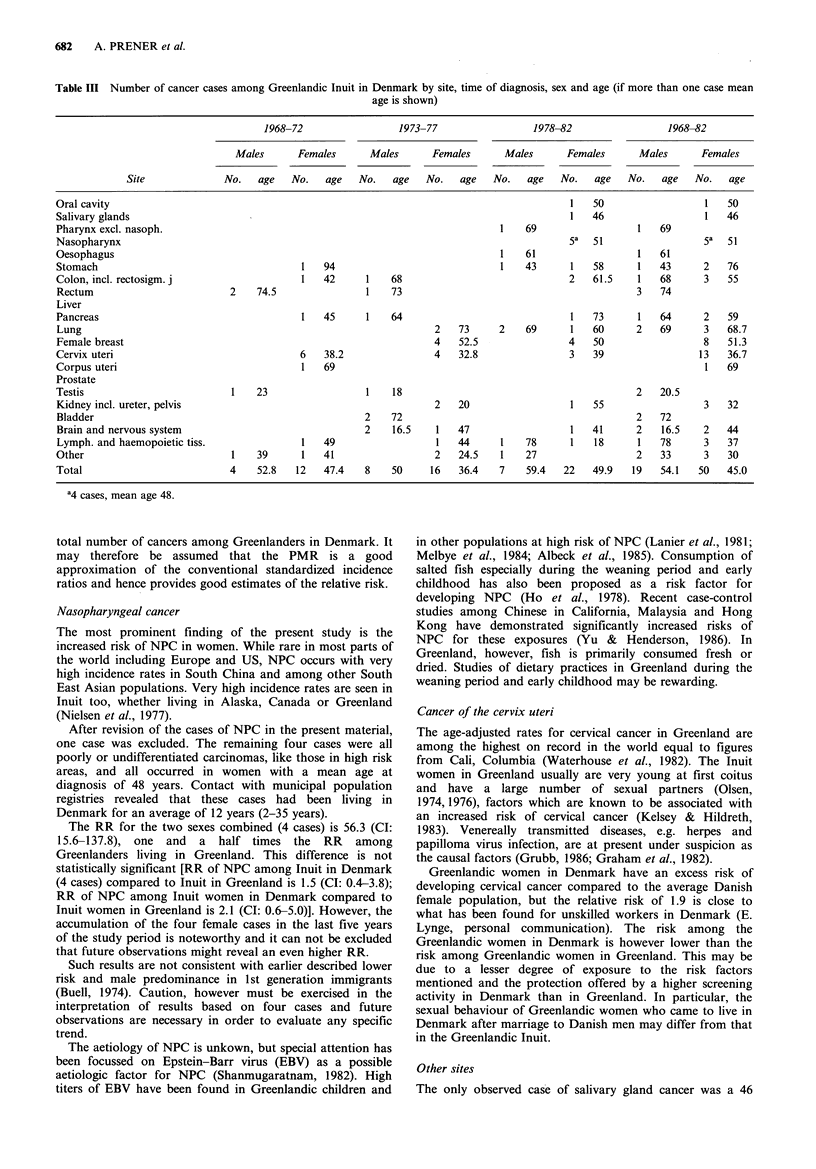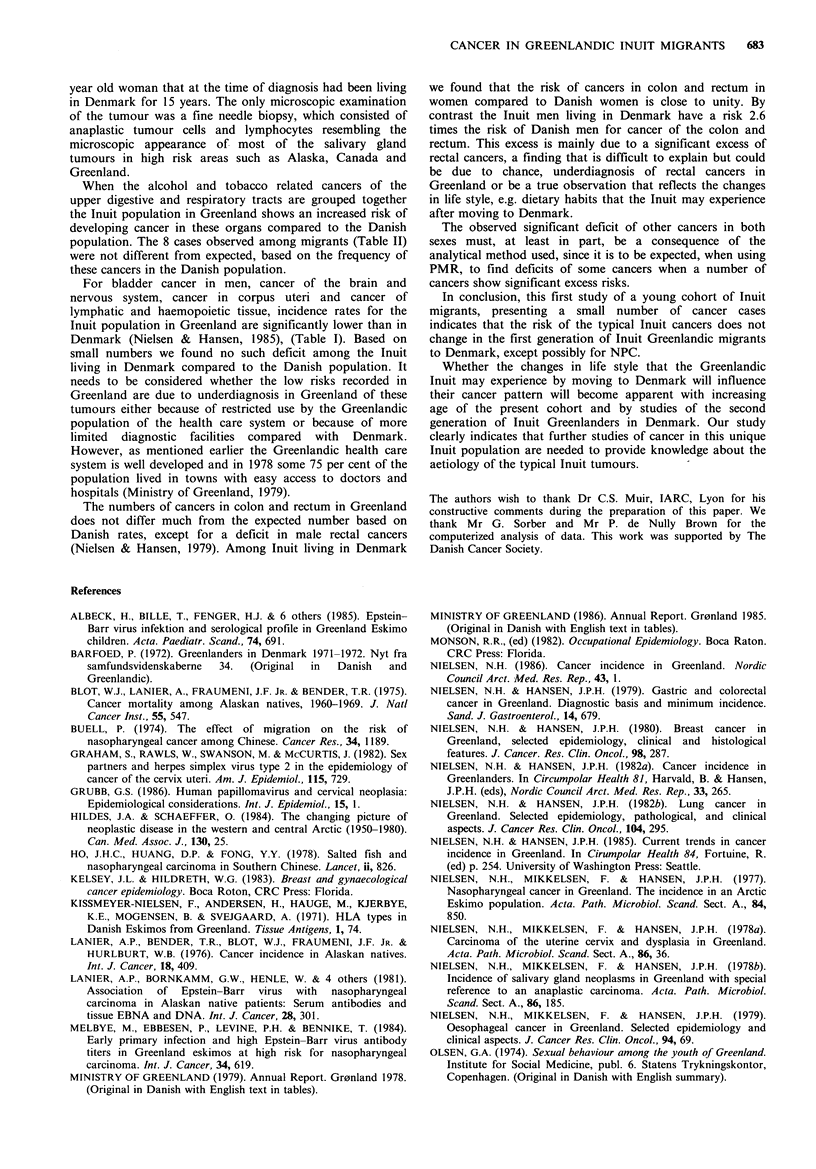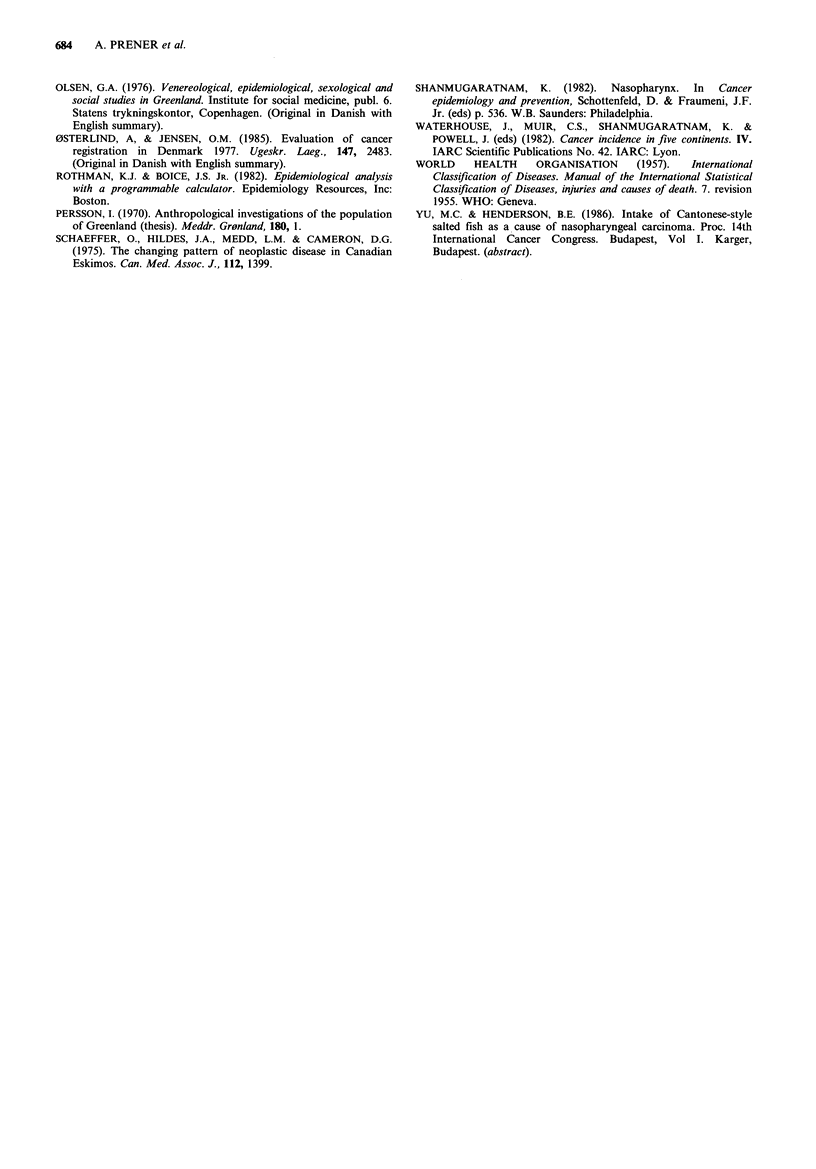# Cancer pattern among Greenlandic Inuit migrants in Denmark, 1968-1982.

**DOI:** 10.1038/bjc.1987.265

**Published:** 1987-11

**Authors:** A. Prener, N. H. Nielsen, J. P. Hansen, O. M. Jensen

**Affiliations:** Danish Cancer Registry, Institute of Cancer Epidemiology under the Danish Cancer Society, Copenhagen.

## Abstract

For several cancer sites the incidence among Inuit (Eskimos) in Alaska, Canada and Greenland differs markedly from that in non-Inuit in adjacent areas. This is the first study of Inuit migrants. Among 11,571 Inuit Greenlandic people living in Denmark in the period 1968-1982 we found 69 cases of cancer. Significantly increased risks compared to the Danish population were found for cancer of the rectum (RR = 5.5) in males and for nasopharyngeal cancer (RR = 185.2) and cancer of the cervix uteri (RR = 1.9). The significance of these findings in relation to the role of environmental factors in the aetiology of cancer in Inuit is discussed.


*Br. J Cancer* (1987), **56**, 679–684 © The Macmillan Press Ltd., 1987

### Cancer pattern among Greenlandic Inuit migrants in Denmark, 1968–1982

A. Prener[1], N.H. Nielsen[2], J.P.H. Hansen[3] & O.M. Jensen[1]

[1]*Danish Cancer Registry, Institute of Cancer Epidemiology under the Danish Cancer Society, Landskronagade 66, DK-2100 Copenhagen;* [2]*Department of Pathology, Rigshospitalet (State University Hospital), Copenhagen and Institute of Forensic Pathology, Copenhagen University, Copenhagen and* [3]*Department of Pathology, Gentofte Hospital, University of Copenhagen, Copenhagen, Denmark.*

**Summary** For several cancer sites the incidence among Inuit (Eskimos) in Alaska, Canada and Greenland differs markedly from that in non-Inuit in adjacent areas. This is the first study of Inuit migrants. Among 11,571 Inuit Greenlandic people living in Denmark in the period 1968–1982 we found 69 cases of cancer. Significantly increased risks compared to the Danish population were found for cancer of the rectum (RR = 5.5) in males and for nasopharyngeal cancer (RR = 185.2) and cancer of the cervix uteri (RR = 1.9). The significance of these findings in relation to the role of environmental factors in the aetiology of cancer in Inuit is discussed.

Inuit (Eskimos) in Alaska, Canada and Greenland have a remarkable cancer pattern and for several sites the incidence differs markedly from that in non-Inuit in adjacent areas (Blot *et al.*, 1975; Nielsen & Hansen, 1982*a*; Lanier *et al.*, 1976; Hildes & Schaeffer, 1984). Studies in Alaska and Canada have in recent years shown a change towards a more westernized cancer pattern (Lanier *et al.*, 1976; Schaeffer *et al.*, 1975). Similar changes have not yet been observed in Greenland (Nielsen & Hansen, 1985) and the Greenlandic population still presents an increased risk for specific types of cancer compared to the population in Denmark. Some of the more prominent findings are the excesses in risk of nasopharyngeal cancer (NPC), cancer of the salivary glands and cancer of the cervix uteri (Nielsen *et al.*, 1977, 1978*a*, *b*). NPC has been related to Epstein–Barr virus infection and migrants from other populations at high risk of NPC have been shown to remain at high risk of NPC even in the second and third generation (Buell, 1974). Cancer of the salivary glands occurs in general with uniform low incidence all over the world except for the Inuit populations in Canada, Alaska and Greenland, and possibly also among the populations of the Doubs, France and in Hawaii (Waterhouse *et al.*, 1982).

This study is concerned with the description of the cancer pattern among the Greenlanders who from the early 1950s have migrated to Denmark and thereby may have changed their life style to a great extent. It is the first study of cancer among Inuit who have migrated to a non-arctic area.

### *Population and health care in Greenland*

Greenland, the worlds largest island, is situated northeast of the mainland of North America. The majority of the population lives on the west coast. The climate is arctic. Greenland's indigenous population is of Inuit origin and constitute the worlds largest group of Inuit. Since the 17th century the Greenlandic Inuit population became strongly intermixed through contact with European whalers and Danish settlers. The present indigenous population of 44,053 (Jan. 1, 1986) (Ministry of Greenland, 1986) shows an average European genetic intermixture of 25–30 per cent, according to blood grouping and tissue (HLA) typing (Kissmeyer-Nielsen *et al.*, 1971; Persson, 1970).

Until the end of the Second World War Greenland was a closed colonial society, and had been a Danish colony for 200 years. In 1953 Greenland became an integral part of the Kingdom of Denmark and this was followed by reforms in economic, administrative and social spheres. In 1979 Home Rule was introduced by which Greenland attained control of its own domestic affairs while remaining within the Kingdom of Denmark.

Health services in Greenland are financed by the Danish state and all medical facilities are free of charge. In 1950 there were 13 hospitals and 15 doctors in Greenland, and a central hospital for all of Greenland was established in the capital Nuuk/Godthåb in 1957. In 1984 there were 16 hospitals and 61 doctors in Greenland. Since the 1950s Danish medical specialists have travelled from district to district during the summer period. A number of patients are transferred each year from the local hospitals to the central hospital in Nuuk and a considerable number of patients (1,075 in 1984) are transferred to hospitals in Denmark for diagnostic purposes and specialized treatment (Ministry of Greenland, 1986).

### *Cancer in Greenland*

Cancer incidence figures for Greenland are available for the period 1950 to 1983 and registration should be virtually complete (Nielsen, 1986; Nielsen & Hansen, 1985). Table I provides an overview of the cancer pattern in the Greenlandic population compared to Denmark for the period, 1975–1983. Excess risks are noted for nasopharyngeal carcinoma, carcinoma in the salivary glands i.e., malignant lymphoepithelial lesions or so called 'eskimomas', and cancer of the oesophagus (Nielsen & Hansen, 1985; Nielsen *et al.*, 1979). Cancers of gastro-intestinal organs do not show large differences in incidence compared to Denmark (Nielsen & Hansen, 1979).

Breast cancer incidence has been increasing since 1950, but is still at a significantly lower level compared to Denmark (Nielsen & Hansen, 1980, 1985). By contrast, the incidence of cervix cancer has increased dramatically and the incidence of cervix cancer in Greenland is today among the highest in the world (1975–83: 64.2 per 100,000 person years, World standard Population (WS)) (Nielsen & Hansen, 1985).

Cancer of the lung has attained an incidence among men which is close to that in Denmark, while the incidence among women is one of the highest on record in the world (1975–83: 21.4 per 100,000 person years (WS)) (Nielsen & Hansen, 1982*b*, 1985).

### Materials and methods

### *Identification of Greenlanders in Denmark*

The Central Population Registry (CPR) in Denmark includes

Correspondence: A. Prener.
Received 5 January 1987; and in revised form, 16 June 1987.

information on all residents in Denmark since April 1, 1968. It is updated daily with changes in the information registered. The CPR comprises information on place of birth, as well as the most recent and up to two previous addresses or the number of addresses in the last 39 months. Changes of address are reported to the CPR by municipal registries, which in turn receive the information from individuals who move within or between municipalities and also on emigration. Since the population in Greenland are residents in the Kingdom of Denmark their movements between Greenland and Denmark are not registered as 'international' migrations, but only as movements between different parts of the Kingdom. For the majority of Greenlanders living in Denmark information on date of entry into Denmark is therefore not easily available.

On June 28, 1985 all persons identifiable in the CPR as being born in Greenland and living in Denmark some time since April 1, 1968 were identified, including persons that died during 1968–1985. Persons that moved back to Greenland and then moved more than twice in the following 39 months could not be identified. Comparison with tables on migrations in Denmark from the Danish Statistical Office estimates this to be a very small proportion, and these persons are not expected to differ from the rest of the cohort in relation to cancer risk.

### *Identification of cancer cases*

A population based national cancer registry was established in Denmark in 1942. Data have been reported from Greenland since around 1970, but these are incomplete and have not been considered for the present analysis. The registration of cancer patients from Denmark is virtually complete (Østerlind & Jensen, 1985). Cancer cases among Greenlanders in Denmark during the time period 1968–82 were identified by a computerized linkage with the Danish Cancer Registry on January 23, 1986. Cases not living permanently in Denmark at the time of diagnosis were excluded. Cancer patients from Greenland who are referred to Denmark for diagnosis and treatment retain their Greenlandic address.

All cases were controlled for ethnic origin either by identification of parents and information on their birthplace or by name (definition of a Greenlandic Inuit: An individual whose father and/or mother was born in Greenland or an individual with a typical Greenlandic family name). Cases that were not of Inuit origin were excluded.

All cases were classified according to The 7th International Classification of Diseases (ICD 7) (World Health Organization, 1957) in accordance with the coding system used in the Danish Cancer Registry since its start.

### *Analysis*

As mentioned earlier the registration of movements by the CPR provides no information on date of entry into Denmark for the majority of Greenlanders living in Denmark. Thus, in the absence of an exact denominator population incidence rates can not be calculated. Relative risks (i.e. observed number divided by expected number) have therefore been estimated as standardized proportional incidence ratios (PMR), with indirect standardization for age (5-year age groups), sex and calendar time (5-year periods). Expected numbers are based on the age- and sex-specific proportional distribution of cancer cases in the Danish population (excluding Greenland) in the same time periods.

Calculation of the 95% confidence intervals for the relative risks based on incidence rate ratios in Table I were done by use of the approximate method by Rothman and Boice (1982), under the assumption that the observed number of cases follows a Poisson distribution and the expected number is constant.

The 95% confidence intervals for the relative risks, estimated as age-standardized proportional incidence rate ratios (Table II), were calculated assuming that the observed number of cases follows a binomial distribution with a total equal to the total number of cancers in the study population and assuming that the expected number is constant.

### Results

A total of 11,571 persons born in Greenland and living in Denmark in the period 1968–1985 were identified from the

**Table I** Observed numbers of cancers among Inuit in Greenland and relative risks compared to the Danish population in Denmark, 1975–83

| | | *Males* | | | *Females* | | | *Both sexes* | | |
|---|---|---|---|---|---|---|---|---|---|---|
| *Site* | *(ICD7)* | *O* | *RR*[a] | *95%CI*[b] | *O* | *RR*[a] | *95%CI*[b] | *O* | *RR*[a] | *95%CI*[b] |
| Oral cavity | (141, 143, 144) | 9 | 4.6 | 2.1 - 8.6 | 0 | 0 | 0 - 2.7 | 9 | 2.7 | 1.2 - 5.1 |
| Salivary glands | (142) | 4 | 6.2 | 1.7 -15.8 | 7 | 13.7 | 5.5 -28.3 | 11 | 9.5 | 4.7 -17.0 |
| Pharynx excl. nasopharynx | (145, 147–148) | 3 | 2.5 | 0.5 - 7.4 | 1 | 1.9 | 0.03–10.7 | 4 | 2.4 | 0.6 - 6.0 |
| Nasopharynx | (146) | 13 | 17.1 | 9.1 -29.3 | 13 | 29.0 | 15.4 -49.4 | 26 | 21.5 | 14.0 -31.5 |
| Oesophagus | (150) | 20 | 5.7 | 3.5 - 8.8 | 13 | 8.3 | 4.4 -14.3 | 33 | 6.5 | 4.5 - 9.1 |
| Stomach | (151) | 10 | 0.6 | 0.3 - 1.2 | 6 | 0.5 | 0.2 - 1.2 | 16 | 0.9 | 0.3 - 0.97 |
| Colon incl. rectosigm. j. | (153) | 14 | 0.8 | 0.4 - 1.3 | 18 | 0.7 | 0.4 - 1.2 | 32 | 0.7 | 0.5 - 1.1 |
| Rectum | (154) | 6 | 0.4 | 0.1 - 0.8 | 11 | 0.8 | 0.4 - 1.5 | 17 | 0.6 | 0.3 - 0.9 |
| Liver | (155) | 6 | 1.8 | 0.7 - 3.9 | 1 | 0.4 | 0.01- 2.3 | 7 | 1.2 | 0.5 - 2.5 |
| Pancreas | (157) | 4 | 0.4 | 0.1 - 1.0 | 10 | 1.2 | 0.6 - 2.2 | 14 | 0.8 | 0.4 - 1.3 |
| Lung | (162) | 57 | 1.1 | 0.8 - 1.4 | 27 | 1.7 | 1.1 - 2.4 | 84 | 1.2 | 0.95- 1.5 |
| Female breast | (170) | – | – | – | 38 | 0.5 | 0.3 - 0.7 | – | – | – |
| Cervix uteri | (171) | – | – | – | 94 | 2.9 | 2.4 - 3.6 | – | – | – |
| Corpus uteri | (172) | – | – | – | 8 | 0.5 | 0.2 - 0.95 | – | – | – |
| Prostate | (177) | 2 | 0.1 | 0.01- 0.3 | – | – | – | – | – | – |
| Testis | (178) | 5 | 0.4 | 0.1 - 0.9 | – | – | – | – | – | – |
| Kidney incl. ureter, pelvis | (180) | 7 | 0.7 | 0.3 - 1.5 | 7 | 1.0 | 0.4 - 2.1 | 14 | 0.8 | 0.5 - 1.4 |
| Bladder | (181) | 9 | 0.4 | 0.2 - 0.7 | 6 | 0.8 | 0.3 - 1.7 | 15 | 0.5 | 0.3 - 0.8 |
| Brain and nervous system | (193) | 4 | 0.3 | 0.08- 0.8 | 6 | 0.5 | 0.2 - 1.1 | 10 | 0.4 | 0.2 - 0.7 |
| Lymph and haemopoietic tiss. | (200–202, 204) | 11 | 0.5 | 0.2 - 0.8 | 9 | 0.5 | 0.2 - 0.9 | 20 | 0.5 | 0.3 - 0.7 |
| Other | | 55 | – | – | 61 | – | – | 116 | – | – |
| Total | | 233 | 0.8 | 0.7 - 0.9 | 335 | 1.0 | 0.9 - 1.1 | 568 | 0.9 | 0.8 - 1.0 |

Source: Nielsen & Hansen, 1985; [a]RR = relative risk = age-standardized incidence rate ratio; [b]95% confidence interval (for details of calculation see text).

**Table II** Observed numbers of cancers among Inuit Greenlanders in Denmark and relative risks compared to the Danish population in Denmark, 1968–82

| Site | (ICD7) | *Males* | | | *Females* | | | *Both sexes* | | |
|---|---|---|---|---|---|---|---|---|---|---|
| | | 0 | RR[a] | 95%CI[b] | 0 | RR[a] | 95%CI[b] | 0 | RR[a] | 95%CI[b] |
| Oral cavity | (141, 143, 144) | 0 | 0 | 0 –35.2 | 1 | 5.5 | 0.1 – 29.4 | 1 | 3.6 | 0.1 – 19.5 |
| Salivary glands | (142) | 0 | 0 | 0 –59.9 | 1 | 9.4 | 0.2 – 49.8 | 1 | 6.1 | 0.2 – 33.1 |
| Pharynx excl. nasopharynx | (145, 147,–148) | 1 | 17.0 | 0.4 –86.8 | 0 | 0 | 0 – 22.8 | 1 | 4.7 | 0.1 – 25.1 |
| Nasopharynx | (146) | 0 | 0 | 0 –76.2 | 5[c] | 185.2 | 61.7 –403.9 | 5[c] | 70.4 | 23.2 –157.1 |
| Oesophagus | (150) | 1 | 6.5 | 0.2 –31.9 | 0 | 0 | 0 – 24.5 | 1 | 3.3 | 0.09– 18.0 |
| Stomach | (151) | 1 | 1.2 | 0.03– 5.9 | 2 | 1.6 | 0.2 – 5.5 | 3 | 1.5 | 0.4 – 4.1 |
| Colon, incl. rectosigm.j. | (153) | 1 | 1.0 | 0.02– 4.9 | 3 | 1.2 | 0.3 – 3.4 | 4 | 1.2 | 0.3 – 2.8 |
| Rectum | (154) | 3 | 5.5 | 1.2 –13.8 | 0 | 0 | 0 – 3.0 | 3 | 1.7 | 0.4 – 4.8 |
| Liver | (155) | 0 | 0 | 0 –22.7 | 0 | 0 | 0 – 13.3 | 0 | 0 | 0 – 8.7 |
| Pancreas | (157) | 1 | 2.1 | 0.05–10.3 | 2 | 2.8 | 0.3 – 9.6 | 3 | 2.5 | 0.5 – 7.0 |
| Lung | (162) | 2 | 0.7 | 0.09– 2.3 | 3 | 1.4 | 0.3 – 3.9 | 5 | 1.0 | 0.3 – 2.3 |
| Female breast | (170) | – | – | – | 8 | 0.6 | 0.3 – 1.1 | – | – | – |
| Cervix uteri | (171) | – | – | – | 13 | 1.9 | 1.1 – 2.9 | – | – | – |
| Corpus uteri | (172) | – | – | – | 1 | 0.4 | 0.01– 1.9 | – | – | – |
| Prostate | (177) | 0 | 0 | 0 – 2.4 | – | – | – | – | – | – |
| Testis | (178) | 2 | 1.2 | 0.2 – 3.7 | – | – | – | – | – | – |
| Kidney incl. ureter, pelvis | (180) | 0 | 0 | 0 – 7.0 | 3 | 3.0 | 0.6 – 8.3 | 3 | 2.0 | 0.4 – 5.7 |
| Bladder | (181) | 2 | 1.6 | 0.2 – 5.0 | 0 | 0 | 0 – 4.5 | 2 | 1.0 | 0.1 – 3.4 |
| Brain and nervous system | (193) | 2 | 1.6 | 0.2 – 4.9 | 2 | 0.8 | 0.1 – 2.9 | 4 | 1.1 | 0.3 – 2.7 |
| Lymph. and haemopoietic tissue | (200–202, 204) | 1 | 0.5 | 0.02– 3.9 | 3 | 1.0 | 0.2 – 2.7 | 4 | 0.8 | 0.2 – 1.9 |
| Other | | 2 | 0.3 | 0.04– 0.99 | 3 | 0.3 | 0.05– 0.7 | 5 | 0.3 | 0.09– 0.6 |
| Total | | 19 | – | – | 50 | – | – | 69 | – | – |

[a]RR = relative risk = age-standardized proportional incidence ratio; [b]95% confidence interval (for details of calculation see text); [c]After revision of diagnosis only 4 cases remained.

CPR. Five thousand one hundred and two (44%) were men and 6,469 (56%) were women. Only 4.9% of the men and 10.1% of the women were born before 1938 and only around 8% were therefore 45 years of age or older in 1982. For comparison the Danish population in 1980 was characterized by 36% being 45 years of age or older and the Inuit population in Greenland by 18% being 45 years or older in 1984.

Altogether 69 cases of cancer were diagnosed at a time when the cohort members were living in Denmark.

Table II shows the number of observed cases and age standardized relative risks by site compared to the Danish population in men and women of Inuit origin born in Greenland and living in Denmark. For comparison age standardized incidence rate-ratios are shown for the same sites for Inuit in Greenland (Table I). In Table III age (or mean age if more than one case) of all cancer cases in Inuit in Denmark is shown by site, 5-year periods and sex.

A total of 19 cases were identified among men from 1968 to 1982. All cases were histologically verified. A significant excess compared to the Danish population in Denmark was observed for cancer of the rectum (RR = 5.5, 95% CI: 1.2–13.8). Non-significant excesses were found for a number of sites, with the highest RR for cancer of the pharynx excluding nasopharynx (RR = 17.0, 95% CI: 0.4–86.8), cancer of the oesophagus (RR = 6.5, 95% CI: 0.2–31.9) and pancreas cancer (RR = 2.1, 95% CI: 0.05–10.3). A marginally significant deficit of other cancers was observed (RR = 0.3, 95% CI: 0.04–0.99). The one case of cancer in haemopoietic and lymphatic tissues was a non-Hodgkin lymphoma in a 78 year old man that moved to Denmark at the age of 20.

Altogether 50 cases were identified in females in the time period 1968–1982. A total of 96% of the cases were histologically verified; 2% (one case) were verified by cytology and 2% (one case) by explorative laparotomy. Significant excesses were found for NPC (RR = 185.2, 95% CI: 61.7–403.9) and cancer of the cervix uteri (RR = 1.9, 95% CI: 1.1–2.9). The five cases of NPC were reviewed by use of the original medical records and one case was found to be an oropharyngeal cancer. When this case was excluded, four cases of NPC in women remained (RR = 148.2, 95% CI: 41.1–356.1). A non-significant increased risk of salivary gland cancer was observed (RR = 9.4, 95% CI: 0.2–49.8). Also in women a significant deficit of other cancers was identified (RR = 0.3, 95% CI: 0.05–0.7), additionally there was a deficit of breast cancer which was not significant (RR = 0.6, 95% CI: 0.3–1.1). The three cases of cancer in haemopoietic and lymphatic tissues were one case of non-Hodgkin lymphoma (RR = 1.2, 95% CI: 0.03–8.3) and two cases of leukaemia (RR = 1.2, 95% CI: 0.3–4.8).

### Discussion

Migration of human populations provides opportunities to study the role of environmental factors in the development of cancer. Useful insights on the aetiology of a number of cancers have been derived from the observation of changes or persistence of risks. Interpretation of the present results is facilitated by the fact that the Inuit population in Greenland is quite uniform in respect to social and economic criteria and no selection bias is likely to affect the risk of cancer in the group that migrated to Denmark compared with the Inuit in Greenland.

For men the two main reasons for leaving Greenland have been improved possibilities for education and employment. Also among women there has been a desire for education, but about half of the Greenlandic women living in Denmark have moved after marriage to a Dane, who then returned to Denmark after an employment period in Greenland (Barfoed, 1972).

Since information on date of entry into Denmark is lacking the denominator population is unknown. When the overall incidence of cancer is similar in the study and the standard population and when the cancers of any single site only constitute a minor part of all malignancies in the study, the PMR is assumed to be similar to SMR (Monson, 1982). The total age-standardized incidence rates are quite similar for the Inuit population in Greenland and for Denmark and no single site of cancer constitutes more than 30% of the

**Table III** Number of cancer cases among Greenlandic Inuit in Denmark by site, time of diagnosis, sex and age (if more than one case mean age is shown)

| | *1968–72* | | | | *1973–77* | | | | *1978–82* | | | | *1968–82* | | | |
|---|---|---|---|---|---|---|---|---|---|---|---|---|---|---|---|---|
| | *Males* | | *Females* | | *Males* | | *Females* | | *Males* | | *Females* | | *Males* | | *Females* | |
| *Site* | *No.* | *age* | *No.* | *age* | *No.* | *age* | *No.* | *age* | *No.* | *age* | *No.* | *age* | *No.* | *age* | *No.* | *age* |
| Oral cavity | | | | | | | | | | | 1 | 50 | | | 1 | 50 |
| Salivary glands | | | | | | | | | | | 1 | 46 | | | 1 | 46 |
| Pharynx excl. nasoph. | | | | | | | | | 1 | 69 | | | 1 | 69 | | |
| Nasopharynx | | | | | | | | | | | 5[a] | 51 | | | 5[a] | 51 |
| Oesophagus | | | | | | | | | 1 | 61 | | | 1 | 61 | | |
| Stomach | | | 1 | 94 | | | | | 1 | 43 | 1 | 58 | 1 | 43 | 2 | 76 |
| Colon, incl. rectosigm. j | | | 1 | 42 | 1 | 68 | | | | | 2 | 61.5 | 1 | 68 | 3 | 55 |
| Rectum | 2 | 74.5 | | | 1 | 73 | | | | | | | 3 | 74 | | |
| Liver | | | | | | | | | | | | | | | | |
| Pancreas | | | 1 | 45 | 1 | 64 | | | | | 1 | 73 | 1 | 64 | 2 | 59 |
| Lung | | | | | | | 2 | 73 | 2 | 69 | 1 | 60 | 2 | 69 | 3 | 68.7 |
| Female breast | | | | | | | 4 | 52.5 | | | 4 | 50 | | | 8 | 51.3 |
| Cervix uteri | | | 6 | 38.2 | | | 4 | 32.8 | | | 3 | 39 | | | 13 | 36.7 |
| Corpus uteri | | | 1 | 69 | | | | | | | | | | | 1 | 69 |
| Prostate | | | | | | | | | | | | | | | | |
| Testis | 1 | 23 | | | 1 | 18 | | | | | | | 2 | 20.5 | | |
| Kidney incl. ureter, pelvis | | | | | | | 2 | 20 | | | 1 | 55 | | | 3 | 32 |
| Bladder | | | | | 2 | 72 | | | | | | | 2 | 72 | | |
| Brain and nervous system | | | | | 2 | 16.5 | 1 | 47 | | | 1 | 41 | 2 | 16.5 | 2 | 44 |
| Lymph. and haemopoietic tiss. | | | 1 | 49 | | | 1 | 44 | 1 | 78 | 1 | 18 | 1 | 78 | 3 | 37 |
| Other | 1 | 39 | 1 | 41 | | | 2 | 24.5 | 1 | 27 | | | 2 | 33 | 3 | 30 |
| Total | 4 | 52.8 | 12 | 47.4 | 8 | 50 | 16 | 36.4 | 7 | 59.4 | 22 | 49.9 | 19 | 54.1 | 50 | 45.0 |

[a]4 cases, mean age 48.

total number of cancers among Greenlanders in Denmark. It may therefore be assumed that the PMR is a good approximation of the conventional standardized incidence ratios and hence provides good estimates of the relative risk.

*Nasopharyngeal cancer*

The most prominent finding of the present study is the increased risk of NPC in women. While rare in most parts of the world including Europe and US, NPC occurs with very high incidence rates in South China and among other South East Asian populations. Very high incidence rates are seen in Inuit too, whether living in Alaska, Canada or Greenland (Nielsen *et al.*, 1977).

After revision of the cases of NPC in the present material, one case was excluded. The remaining four cases were all poorly or undifferentiated carcinomas, like those in high risk areas, and all occurred in women with a mean age at diagnosis of 48 years. Contact with municipal population registries revealed that these cases had been living in Denmark for an average of 12 years (2–35 years).

The RR for the two sexes combined (4 cases) is 56.3 (CI: 15.6–137.8), one and a half times the RR among Greenlanders living in Greenland. This difference is not statistically significant [RR of NPC among Inuit in Denmark (4 cases) compared to Inuit in Greenland is 1.5 (CI: 0.4–3.8); RR of NPC among Inuit women in Denmark compared to Inuit women in Greenland is 2.1 (CI: 0.6–5.0)]. However, the accumulation of the four female cases in the last five years of the study period is noteworthy and it can not be excluded that future observations might reveal an even higher RR.

Such results are not consistent with earlier described lower risk and male predominance in 1st generation immigrants (Buell, 1974). Caution, however must be exercised in the interpretation of results based on four cases and future observations are necessary in order to evaluate any specific trend.

The aetiology of NPC is unkown, but special attention has been focussed on Epstein–Barr virus (EBV) as a possible aetiologic factor for NPC (Shanmugaratnam, 1982). High titers of EBV have been found in Greenlandic children and in other populations at high risk of NPC (Lanier *et al.*, 1981; Melbye *et al.*, 1984; Albeck *et al.*, 1985). Consumption of salted fish especially during the weaning period and early childhood has also been proposed as a risk factor for developing NPC (Ho *et al.*, 1978). Recent case-control studies among Chinese in California, Malaysia and Hong Kong have demonstrated significantly increased risks of NPC for these exposures (Yu & Henderson, 1986). In Greenland, however, fish is primarily consumed fresh or dried. Studies of dietary practices in Greenland during the weaning period and early childhood may be rewarding.

*Cancer of the cervix uteri*

The age-adjusted rates for cervical cancer in Greenland are among the highest on record in the world equal to figures from Cali, Columbia (Waterhouse *et al.*, 1982). The Inuit women in Greenland usually are very young at first coitus and have a large number of sexual partners (Olsen, 1974, 1976), factors which are known to be associated with an increased risk of cervical cancer (Kelsey & Hildreth, 1983). Venereally transmitted diseases, e.g. herpes and papilloma virus infection, are at present under suspicion as the causal factors (Grubb, 1986; Graham *et al.*, 1982).

Greenlandic women in Denmark have an excess risk of developing cervical cancer compared to the average Danish female population, but the relative risk of 1.9 is close to what has been found for unskilled workers in Denmark (E. Lynge, personal communication). The risk among the Greenlandic women in Denmark is however lower than the risk among Greenlandic women in Greenland. This may be due to a lesser degree of exposure to the risk factors mentioned and the protection offered by a higher screening activity in Denmark than in Greenland. In particular, the sexual behaviour of Greenlandic women who came to live in Denmark after marriage to Danish men may differ from that in the Greenlandic Inuit.

*Other sites*

The only observed case of salivary gland cancer was a 46

year old woman that at the time of diagnosis had been living in Denmark for 15 years. The only microscopic examination of the tumour was a fine needle biopsy, which consisted of anaplastic tumour cells and lymphocytes resembling the microscopic appearance of most of the salivary gland tumours in high risk areas such as Alaska, Canada and Greenland.

When the alcohol and tobacco related cancers of the upper digestive and respiratory tracts are grouped together the Inuit population in Greenland shows an increased risk of developing cancer in these organs compared to the Danish population. The 8 cases observed among migrants (Table II) were not different from expected, based on the frequency of these cancers in the Danish population.

For bladder cancer in men, cancer of the brain and nervous system, cancer in corpus uteri and cancer of lymphatic and haemopoietic tissue, incidence rates for the Inuit population in Greenland are significantly lower than in Denmark (Nielsen & Hansen, 1985), (Table I). Based on small numbers we found no such deficit among the Inuit living in Denmark compared to the Danish population. It needs to be considered whether the low risks recorded in Greenland are due to underdiagnosis in Greenland of these tumours either because of restricted use by the Greenlandic population of the health care system or because of more limited diagnostic facilities compared with Denmark. However, as mentioned earlier the Greenlandic health care system is well developed and in 1978 some 75 per cent of the population lived in towns with easy access to doctors and hospitals (Ministry of Greenland, 1979).

The numbers of cancers in colon and rectum in Greenland does not differ much from the expected number based on Danish rates, except for a deficit in male rectal cancers (Nielsen & Hansen, 1979). Among Inuit living in Denmark we found that the risk of cancers in colon and rectum in women compared to Danish women is close to unity. By contrast the Inuit men living in Denmark have a risk 2.6 times the risk of Danish men for cancer of the colon and rectum. This excess is mainly due to a significant excess of rectal cancers, a finding that is difficult to explain but could be due to chance, underdiagnosis of rectal cancers in Greenland or be a true observation that reflects the changes in life style, e.g. dietary habits that the Inuit may experience after moving to Denmark.

The observed significant deficit of other cancers in both sexes must, at least in part, be a consequence of the analytical method used, since it is to be expected, when using PMR, to find deficits of some cancers when a number of cancers show significant excess risks.

In conclusion, this first study of a young cohort of Inuit migrants, presenting a small number of cancer cases indicates that the risk of the typical Inuit cancers does not change in the first generation of Inuit Greenlandic migrants to Denmark, except possibly for NPC.

Whether the changes in life style that the Greenlandic Inuit may experience by moving to Denmark will influence their cancer pattern will become apparent with increasing age of the present cohort and by studies of the second generation of Inuit Greenlanders in Denmark. Our study clearly indicates that further studies of cancer in this unique Inuit population are needed to provide knowledge about the aetiology of the typical Inuit tumours.

The authors wish to thank Dr C.S. Muir, IARC, Lyon for his constructive comments during the preparation of this paper. We thank Mr G. Sorber and Mr P. de Nully Brown for the computerized analysis of data. This work was supported by The Danish Cancer Society.

### References

ALBECK, H., BILLE, T., FENGER, H.J. & 6 others (1985). Epstein–Barr virus infektion and serological profile in Greenland Eskimo children. *Acta. Paediatr. Scand.*, **74**, 691.

BARFOED, P. (1972). Greenlanders in Denmark 1971–1972. Nyt fra samfundsvidenskaberne 34. (Original in Danish and Greenlandic).

BLOT, W.J., LANIER, A., FRAUMENI, J.F. Jr. & BENDER, T.R. (1975). Cancer mortality among Alaskan natives, 1960–1969. *J. Natl Cancer Inst.*, **55**, 547.

BUELL, P. (1974). The effect of migration on the risk of nasopharyngeal cancer among Chinese. *Cancer Res.*, **34**, 1189.

GRAHAM, S., RAWLS, W., SWANSON, M. & McCURTIS, J. (1982). Sex partners and herpes simplex virus type 2 in the epidemiology of cancer of the cervix uteri. *Am. J. Epidemiol.*, **115**, 729.

GRUBB, G.S. (1986). Human papillomavirus and cervical neoplasia: Epidemiological considerations. *Int. J. Epidemiol.*, **15**, 1.

HILDES, J.A. & SCHAEFFER, O. (1984). The changing picture of neoplastic disease in the western and central Arctic (1950–1980). *Can. Med. Assoc. J.*, **130**, 25.

HO, J.H.C., HUANG, D.P. & FONG, Y.Y. (1978). Salted fish and nasopharyngeal carcinoma in Southern Chinese. *Lancet*, **ii**, 826.

KELSEY, J.L. & HILDRETH, W.G. (1983). *Breast and gynaecological cancer epidemiology*. Boca Roton, CRC Press: Florida.

KISSMEYER-NIELSEN, F., ANDERSEN, H., HAUGE, M., KJERBYE, K.E., MOGENSEN, B. & SVEJGAARD, A. (1971). HLA types in Danish Eskimos from Greenland. *Tissue Antigens*, **1**, 74.

LANIER, A.P., BENDER, T.R., BLOT, W.J., FRAUMENI, J.F. Jr. & HURLBURT, W.B. (1976). Cancer incidence in Alaskan natives. *Int. J. Cancer*, **18**, 409.

LANIER, A.P., BORNKAMM, G.W., HENLE, W. & 4 others (1981). Association of Epstein–Barr virus with nasopharyngeal carcinoma in Alaskan native patients: Serum antibodies and tissue EBNA and DNA. *Int. J. Cancer*, **28**, 301.

MELBYE, M., EBBESEN, P., LEVINE, P.H. & BENNIKE, T. (1984). Early primary infection and high Epstein–Barr virus antibody titers in Greenland eskimos at high risk for nasopharyngeal carcinoma. *Int. J. Cancer*, **34**, 619.

MINISTRY OF GREENLAND (1979). Annual Report. Grønland 1978. (Original in Danish with English text in tables).

MINISTRY OF GREENLAND (1986). Annual Report. Grønland 1985. (Original in Danish with English text in tables).

MONSON, R.R., (ed) (1982). *Occupational Epidemiology*. Boca Raton. CRC Press: Florida.

NIELSEN, N.H. (1986). Cancer incidence in Greenland. *Nordic Council Arct. Med. Res. Rep.*, **43**, 1.

NIELSEN, N.H. & HANSEN, J.P.H. (1979). Gastric and colorectal cancer in Greenland. Diagnostic basis and minimum incidence. *Sand. J. Gastroenterol.*, **14**, 679.

NIELSEN, N.H. & HANSEN, J.P.H. (1980). Breast cancer in Greenland, selected epidemiology, clinical and histological features. *J. Cancer. Res. Clin. Oncol.*, **98**, 287.

NIELSEN, N.H. & HANSEN, J.P.H. (1982*a*). Cancer incidence in Greenlanders. In *Circumpolar Health 81*, Harvald, B. & Hansen, J.P.H. (eds), *Nordic Council Arct. Med. Res. Rep.*, **33**, 265.

NIELSEN, N.H. & HANSEN, J.P.H. (1982*b*). Lung cancer in Greenland. Selected epidemiology, pathological, and clinical aspects. *J. Cancer Res. Clin. Oncol.*, **104**, 295.

NIELSEN, N.H. & HANSEN, J.P.H. (1985). Current trends in cancer incidence in Greenland. In *Cirumpolar Health 84*, Fortuine, R. (ed) p. 254. University of Washington Press: Seattle.

NIELSEN, N.H., MIKKELSEN, F. & HANSEN, J.P.H. (1977). Nasopharyngeal cancer in Greenland. The incidence in an Arctic Eskimo population. *Acta. Path. Microbiol. Scand.* Sect. A., **84**, 850.

NIELSEN, N.H., MIKKELSEN, F. & HANSEN, J.P.H. (1978*a*). Carcinoma of the uterine cervix and dysplasia in Greenland. *Acta. Path. Microbiol. Scand.* Sect. A., **86**, 36.

NIELSEN, N.H., MIKKELSEN, F. & HANSEN, J.P.H. (1978*b*). Incidence of salivary gland neoplasms in Greenland with special reference to an anaplastic carcinoma. *Acta. Path. Microbiol. Scand.* Sect. A., **86**, 185.

NIELSEN, N.H., MIKKELSEN, F. & HANSEN, J.P.H. (1979). Oesophageal cancer in Greenland. Selected epidemiology and clinical aspects. *J. Cancer Res. Clin. Oncol.*, **94**, 69.

OLSEN, G.A. (1974). *Sexual behaviour among the youth of Greenland*. Institute for Social Medicine, publ. 6. Statens Trykningskontor, Copenhagen. (Original in Danish with English summary).

OLSEN, G.A. (1976). *Venereological, epidemiological, sexological and social studies in Greenland.* Institute for social medicine, publ. 6. Statens trykningskontor, Copenhagen. (Original in Danish with English summary).

ØSTERLIND, A, & JENSEN, O.M. (1985). Evaluation of cancer registration in Denmark 1977. *Ugeskr. Laeg.*, **147**, 2483. (Original in Danish with English summary).

ROTHMAN, K.J. & BOICE, J.S. JR. (1982). *Epidemiological analysis with a programmable calculator*. Epidemiology Resources, Inc: Boston.

PERSSON, I. (1970). Anthropological investigations of the population of Greenland (thesis). *Meddr. Grønland*, **180,** 1.

SCHAEFFER, O., HILDES, J.A., MEDD, L.M. & CAMERON, D.G. (1975). The changing pattern of neoplastic disease in Canadian Eskimos. *Can. Med. Assoc. J.*, **112,** 1399.

SHANMUGARATNAM, K. (1982). Nasopharynx. In *Cancer epidemiology and prevention,* Schottenfeld, D. & Fraumeni, J.F. Jr. (eds) p. 536. W.B. Saunders: Philadelphia.

WATERHOUSE, J., MUIR, C.S., SHANMUGARATNAM, K. & POWELL, J. (eds) (1982). *Cancer incidence in five continents.* **IV.** IARC Scientific Publications No. 42. IARC: Lyon.

WORLD HEALTH ORGANISATION (1957). *International Classification of Diseases. Manual of the International Statistical Classification of Diseases, injuries and causes of death.* 7. revision 1955. WHO: Geneva.

YU, M.C. & HENDERSON, B.E. (1986). Intake of Cantonese-style salted fish as a cause of nasopharyngeal carcinoma. Proc. 14th International Cancer Congress. Budapest, Vol I. Karger, Budapest. (*abstract*).